# Clinical study on the safety and feasibility of AiWalker-K for lower limbs exercise rehabilitation in children with cerebral palsy

**DOI:** 10.1371/journal.pone.0303517

**Published:** 2024-05-22

**Authors:** Yi Zhang, Zhichong Hui, Weihang Qi, Jiamei Zhang, Mingmei Wang, Dengna Zhu

**Affiliations:** 1 Department of Rehabilitation Medicine, the Third Affiliated Hospital of Zhengzhou University, Zhengzhou, China; 2 Henan Key Laboratory of Child Brain Injury and Henan Pediatric Clinical Research Center, Institute of Neuroscience and Third Affiliated Hospital of Zhengzhou University, Zhengzhou, China; IRCCS Medea: Istituto di Ricovero e Cura a Carattere Scientifico Eugenio Medea, ITALY

## Abstract

**Background:**

Robotic-assisted gait training (RAGT) devices are effective for children with cerebral palsy (CP). Many RAGT devices have been created and put into clinical rehabilitation treatment. Therefore, we aimed to investigate the safety and feasibility of a new RAGT for children with CP.

**Methods:**

This study is a cross-over design with 23 subjects randomly divided into two groups. The occurrence of adverse events and changes in heart rate and blood pressure were recorded during each AiWalker-K training. Additionally, Gross Motor Function Measure-88 (GMFM-88), Pediatric Balance Scale (PBS), 6 Minutes Walking Test (6MWT), Physiological Cost Index, and Edinburgh Visual Gait Score (EVGS) were used to assess treatment, period, carry-over, and follow-up effects in this study.

**Results:**

Adverse events included joint pain, skin pain, and injury. Heart rate and blood pressure were higher with the AiWalker-K compared to the rest (P < 0.05), but remained within safe ranges. After combined treatment with AiWalker-K and routine rehabilitation treatment, significant improvements in 6MWT, GMFM-88 D and E, PBS, and EVGS were observed compared to routine rehabilitation treatment alone (P < 0.05).

**Conclusions:**

Under the guidance of experienced medical personnel, AiWalker-K can be used for rehabilitation in children with CP.

## Introduction

The prevalence of cerebral palsy (CP) is 2–3 per 1,000 live births, but has remained relatively stable over decades [1]. CP is a group of permanent central motor and posture developmental disorders, and activity limitation syndromes caused by non-progressive damage to the brain during development. It is usually accompanied by sensory, perceptual, cognitive, communication, behavioral disturbances, and epilepsy [2]. The primary manifestations of CP are dyskinesias and postural abnormalities. Children with CP often have a reduced ability to walk due to dyskinesias and abnormal posture [3].

With the rapid development of robotic-assisted gait training (RAGT) in recent years, it has become an increasingly common rehabilitation method to improve gait pattern in children with CP [4]. The improvement in the walking ability of lower limbs in children with CP through RAGT mainly depends on the principle of neuroplasticity. Neuroplasticity refers to the adaptive change and remodeling ability of the nervous system to external and internal stimuli, and is a mechanism for the brain and nervous system to adapt to environmental changes [5]. During the RAGT rehabilitation process, the RAGT device monitors and adjusts the patient’s movement through sensors, motors, and other technologies to help the patient perform rehabilitation training. This training can stimulate the neurons in the patient’s brain to generate new synaptic connections, enhance communication between neurons, and increase neuroplasticity [6]. Furthermore, children’s brains are still in the developmental stage and have greater neuroplasticity than adult brains [7]. RAGT can also enhance children’s lower limbs muscle strength, improve gait posture and increase their confidence in further rehabilitation [8].

Mainstream RAGT devices, such as the Lokomat, have been widely used in the field of rehabilitation. Although many studies have confirmed the effectiveness of the Lokomat in the rehabilitation of children with CP [9, 10], the Lokomat is treadmill-based and stationary, redundancy of games, and also has drawbacks such as a large footprint, expensive price, high maintenance costs, and cumbersome operations [11]. Furthermore, a single RAGT device, or even multiple robots with the same rehabilitation principles, may not be able to fully address the needs of all children with CP [12]. At the same time, the use of RAGT devices may have different psychosocial effects on patients [13].

AiWalker-K (Ai-Robotics Technology Co. LTD, Beijing, China) is a passive RAGT device designed for children, with dimensions of 870 mm in length, 825 mm in width, and 1140 mm in height. It is suitable for children with a height of 80 cm to 150 cm, a weight of ≤ 70 kg, and lower limbs motor dysfunction. Its main features are low price, small footprint, easy operation and mobility. It can be easily moved to different locations, and children with lower limbs motor dysfunction can be transferred from a wheelchair to the AiWalker-K in a sitting position, and then adjusted to a standing position after securing the shoulder straps, either in the air or on the real ground modifying the gait cycle. The subjects can see the changing external environment, which enriches the input of visual signals, increases the enjoyment of treatment, and helps to improve their psychological conditions.

Although the AiWalker-K is designed for the rehabilitation of children with lower limbs motor dysfunction also includes children with CP, the safety and effectiveness of the device for the rehabilitation of children with CP and whether it can be used to replace the Lokomat for rehabilitation exercises in children with CP are still unknown. The purpose of this clinical trial is to evaluate the safety and effectiveness of the AiWalker-K as a rehabilitation device for lower limb exercise in children with CP.

## Materials and methods

This study was approved by the Ethics Review Committee of the Third Affiliated Hospital of Zhengzhou University (Number: 2022-406-01) and was registered at the chictr.org.cn (identifier: ChiCTR2300067966). Before the study, the principal investigator (PI) obtained written informed consent from all legal guardians of children with CP.

### Subjects

The participants of this study were children with CP (aged between 3 and 10 years) who were admitted to the Department of Rehabilitation Medicine of the Third Affiliated Hospital of Zhengzhou University between January 2023 and June 2023. The inclusion criteria were: 1) diagnosed with CP; 2) GMFCS level II-IV; 3) height between 80 cm and 150 cm, weight ≤ 70kg; 4) able to reliably report pain, fear, and discomfort; 5) able to understand instructions and cooperate with training; 6) not having received lower limbs surgery or lower limbs botulinum toxin injection in the past 6 months; and 7) drug therapy must be stable for at least 1 month before enrollment in the study. Exclusion criteria were: 1) severe lower limbs contractures; 2) complications of major organs such as the heart, lungs, liver, and kidneys; 3) accompanying severe epilepsy, genetic metabolic diseases, or severe skeletal system diseases; 4) damage or infection of the skin in contact with the device; 5) inflammation in the lower extremities and limited joint movement; 6) participation in other clinical trials of lower limbs rehabilitation within 1 month before this study; and 7) current use of antispasticity medications.

**Fig 1:**
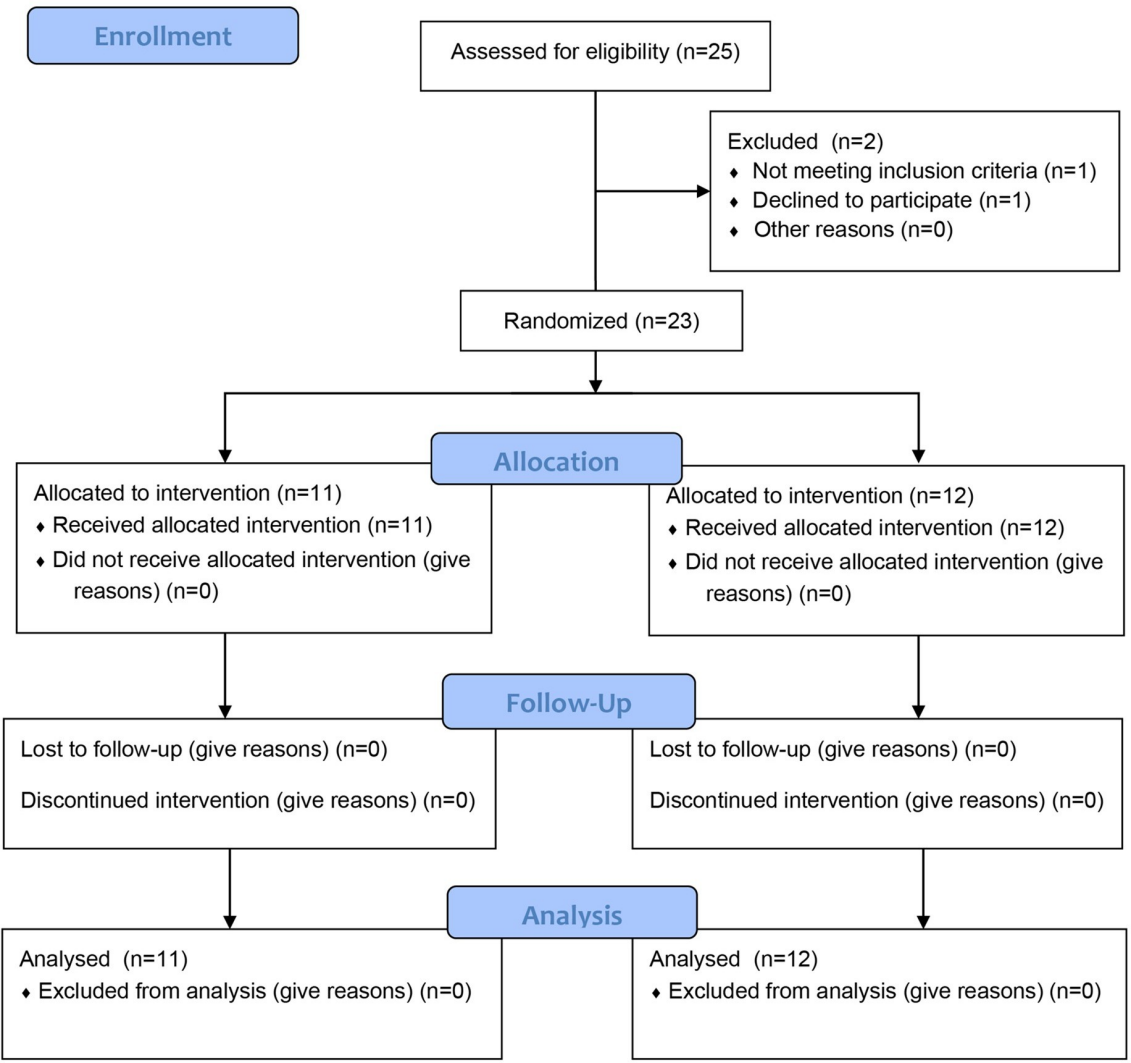
CONSORT 2010 flow diagram. CONSORT flow diagram template courtesy of http://www.consort-statement.org/consort-statement/flow-diagram.

A total of 25 children with CP participated in the study. One child did not meet the inclusion criteria, and one child refused to participate in the study. Consequently, 23 children were randomly assigned to complete the experiment. The Consort flow diagram is shown in Fig 1.

### RAGT device

AiWalker-K is mainly composed of a central control system, four joint driving motors, and a mobile platform, as shown in S1–S3 Figs. The control system outputs motion gait and posture commands to the drive motors, the four driving motors, which perform synchronous and coordinated movements after receiving commands. The control system adjusts the motion parameters of the four driving motors in real time based on state information such as the angular velocity of the four driving motor shafts. The rotation centers of the bilateral hip joints and knee joint motors in the driving motors are on the same horizontal axis as the subjects’ hip joints and knee joint rotation centers, The lower limbs of the device are adjustable and can be quickly adapted to the subjects of different heights. Additionally, the initial angle formed by the rotation center of the bilateral hip joint and knee joint motors and the thigh rod can be adjusted. The mobile platform features an armrest and four wheels for easy mobility, and the device is attached to the wearer’s extremities via straps at the waist, thighs, calves, and feet. The distance between the two movable connecting sections of the lumbar support can be adjusted in width using the width knob, and it is suitable for subjects with different waist widths. When the lower limbs are moving, the hydraulic device can drive the lumbar support device to move up and down relative to the predetermined position. The gait cycle of the device can be adjusted from 2.45 seconds to 5.25 seconds as required. More information about the AiWalker-K can be found in this literature [14].

### Procedures

#### Preparation before experiment

Before using the AiWalker-K, researchers should measure the subjects’ pelvic width, thigh and calf lengths, bilateral hip and knee joint initial angles, and bilateral hip and knee joint mobility, and then adjust the device accordingly. Once the subjects are in the RAGT device, researchers should adjust the RAGT until the soles of the subjects’ feet are off the ground and in a state of full weightlessness and set the gait cycle to a maximum of 5.25 seconds before turning on the RAGT. The researchers should then passively move the subject’s lower limbs under the drive of the RAGT device and gradually adjust the gait cycle to the optimum level. The information of the subjects should be recorded in a register. Researchers should adjust the RAGT device to enable the subjects to walk on the experimental site according to the experimental time to increase the mechanical feedback of the subjects’ plantar. During this period, researchers should observe for any discomfort in the subjects and address it immediately. The most important point is to help the patient overcome their fear of using the AiWalker-K and become familiar with its use during this period to facilitate smooth training of the experimental phase.

#### Experiment

This was a single-center, single-blind, randomized, cross-over study. Both AiWalker-K (A) training and routine rehabilitation (R) training were required to be applied to the subjects. The subjects were randomly divided into Group 1 and Group 2. Group 1 underwent three stages of treatment. The first stage was AiWalker-K combined with routine rehabilitation treatment; the second was routine rehabilitation treatment; and the third was routine rehabilitation treatment (AR/R/R sequence). Group 2 underwent three stages of treatment. The first stage was routine rehabilitation treatment; the second was AiWalker-K combined with routine rehabilitation treatment; and the third was routine rehabilitation treatment (R/AR/R sequence). Efficacy indicators of All subjects were evaluated at baseline and after each training stage.

AiWalker-K training was performed five times a week for four weeks, with each session lasting 30 minutes. The subjects were required to be in a quiet state 10 minutes before the training. The gait cycle should be adjusted to the most suitable setting for each subject and cannot be changed afterward. Rehabilitation therapists ensured the safety and walking direction of the subjects, while family members can provide encouragement and support to the subjects.

All subjects continued their routine rehabilitation treatment during the experimental phase, with no change in the type or frequency of intervention throughout the experiment. The routine rehabilitation treatment includes the following: PhysicalTraining, which includes training in neuromuscular facilitation techniques, balance training, trunk and joint strength training, task-oriented postural control, and movement control training. Each training session lasts 30 minutes and is conducted 10 times per week. Paraffin Therapy: Using the wax cake method, it is applied to the triceps of the calf according to the anatomical position. Each session lasts 30 minutes and is performed five times a week. Neuromuscular electrical stimulation stimulates the antagonist muscles of the triceps brachii and the anterior tibial muscles in the calf. The frequency is set at 1 Hz and the pulse duration is 100 milliseconds. Each treatment lasts 30 minutes and is given five times a week. Tuina therapy is performed by well-trained massage therapists. Each treatment lasts 30 minutes and is given five times a week.

### Observational indicators

#### Safety indicators

Adverse events: Adverse events such as skin pain, skin damage, falls, joint damage, and fractures were recorded while the subjects were using the RAGT device.

Heart rate changes: Heart rates were recorded during “resting state (RS)” and “RAGT” with Polar H10 heart rate chest strap [15]. “RS” refers to the heart rate measured 1 minute before the start of the experiment, while “RAGT” refers to the heart rate measured during the training period between 10 and 20 minutes.

Blood pressure changes: The blood pressure of the left upper limbs was measured twice using a medical electronic blood pressure monitor. The first measurement was taken while the subject was in a seated position immediately before the start of the experiment. The second measurement was taken 3 minutes after the end of the RAGT training.

#### Effectivity indicators

6 Minutes Walking Test (6MWT): The 6MWT is a commonly used test to assess walking endurance in children with CP. During the test, the subjects walk for 6 minutes on a round-trip 50-meter trail at an appropriate speed. Subjects are allowed to take breaks when they feel unable to continue. Researchers record the total walking distance and assistive device used, and each subject should use the same assistive device throughout the test [16]. 6MWT is the main indicator of all effectiveness indicators.

Gross Motor Function Measure-88 (GMFM-88): The GMFM-88 is currently the most widely used scale for assessing gross motor function in children with CP. The scale is divided into five functional areas: Area A includes lying down and turning over (17 items), Area B includes sitting (20 items), Area C includes crawling and kneeling (14 items), Area D includes standing (13 items), and Area E includes walking, running, and jumping (24 items) [17].

Pediatric Balance Scale (PBS): The PBS is used to assess the balance ability of children with mild to moderate motor dysfunction. The scale consists of 14 items, with a total of 56 points, and has been shown to have good reliability [18].

Physiological Cost Index (PCI): The PCI is a commonly used, simple, and feasible index for measuring energy expenditure during walking [19]. Before the test, the subjects were asked to rest quietly in a seated position for 5 minutes, and their resting heart rate was measured. Next, the subjects were asked to walk on a 50-meter trail at their preferred pace for 6 minutes, and the walking distance was measured. Immediately after walking, the subjects rested quietly in their seats, and their heart rate was measured again. The PCI was calculated using the following formula: PCI = (heart rate during walking—heart rate at rest) / walking speed (m/min). A higher PCI indicates a higher energy expenditure of walking per unit of time.

Edinburgh Visual Gait Score (EVGS): EVGS is an effective and reliable clinical evaluation tool for 2D visual gait-posture analysis in children with CP. The EVGS has 17 test items with a total of 68 points [20, 21]. During the test, the subjects walk at an optimal speed on an 8-meter-long trail, while two smartphones with high-speed camera functions are placed at one end of the trail and in the middle to collect the walking data of the children with CP [22]. The walking data is then processed and analyzed using Kinovea motion video analysis software, which has been shown to have good reliability in motion video analysis [23].

Three evaluators with more than five years of work experience were selected to conduct the evaluations. The mean value of their assessments was used, and the evaluators were not informed of the interventions and sequence of each subject.

### Sampling

The sample size was calculated comprehensively using minimal clinically important difference (MCID) literature related to RAGT and combined with the sample size calculation formula of the cross-over trial [24, 25]. The researchers detect the MCID in efficacy indicators under 90% efficacy conditions (efficacy ratio = 0.05), requiring approximately 12 people per group. Based on clinical research experience, a withdrawal rate of 15% was anticipated due to efficacy, compliance, adverse reactions, or other unforeseen factors, meaning that there needed to be at least 14 subjects in each group. Since this study adopts a cross-over design trial, which is more efficient than parallel randomized controlled experiments, the effective sample size is equivalent to 2 times the actual sample size [26]. Therefore, a sample size of 23 is sufficient to meet the experimental requirements.

### Statistical analysiss

Statistical analyses were performed using SPSS 24.0 (IBM Corporation, Armonk, NY, USA). We followed the statistical analysis referenced from the protocol we referenced, we analyzed the data of four effects (treatment, period, carry-over, and follow-up effects) to assess the effects of the RAGT period on 6MWT, GMFM-88, PBS, PCI, and EVGS.

**Fig 2:**
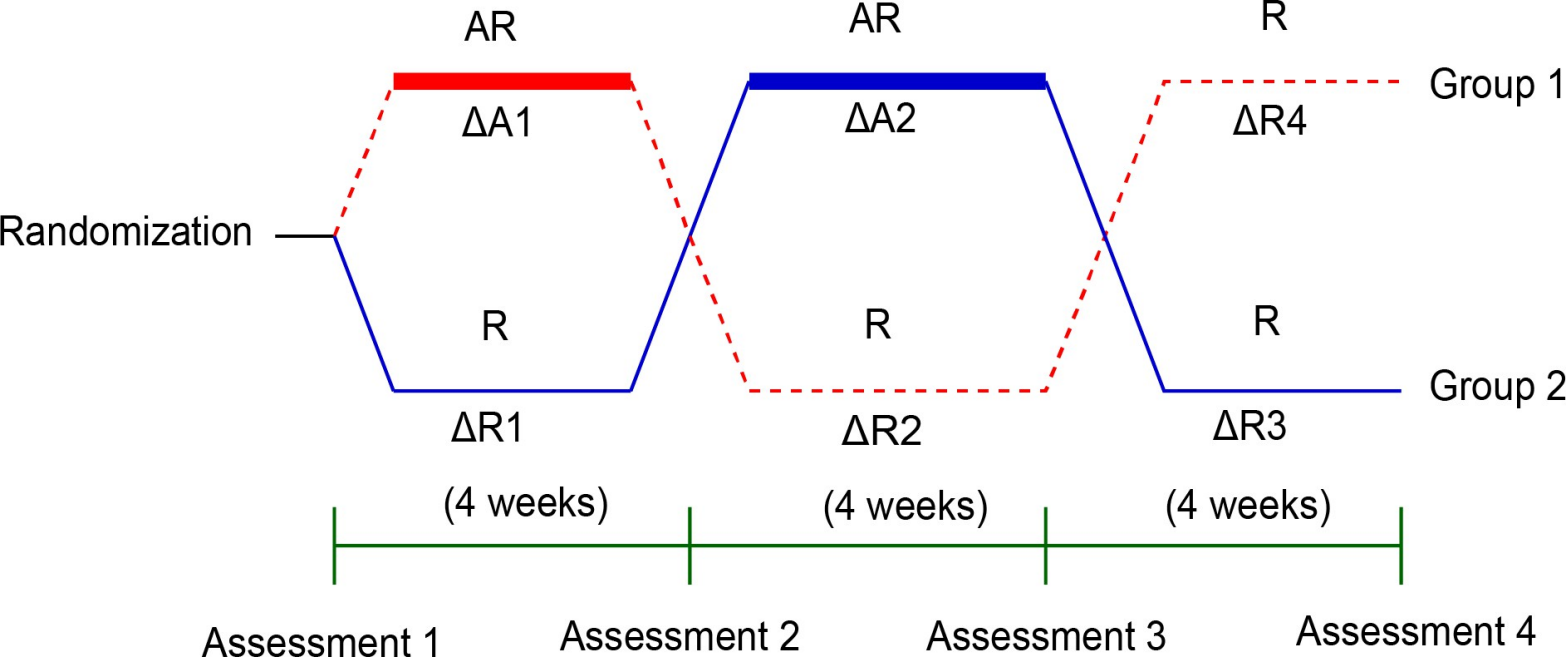
Flow diagram of the study and statistical analysis. AR: AiWalker-K combined with routine rehabilitation treatment; R: routine rehabilitation treatment. The training sequence of Group 1 was AR → R → R, while the training sequence of Group 2 was R → AR → R. Delta (Δ) represents the increment. The training content for ΔA1 and ΔA2 included AiWalker-K training (A) combined with routine rehabilitation training (R), while the training content for ΔR1, ΔR2, ΔR3, and ΔR4 was routine rehabilitation training (R).

Treatment effects were compared using delta (Δ) values obtained during AiWalker-K (A) and routine rehabilitation training (R) (ΔA1 grouped with ΔA2 vs. ΔR1 grouped with ΔR2). Period effects were compared using the Δ values obtained during the first and second training sessions (ΔA1 grouped with ΔR1 vs. ΔA2 grouped with ΔR2). Carry-over effects were compared using the sum of the delta (Δ) values (ΔA1+ΔR2 vs. ΔR1+ΔA2) for the first two periods between Group 1 and Group 2. Finally, follow-up effects were compared using a fourth assessment of Group 1 and Group 2. Between-group differences in heart rate, blood pressure, baseline, and follow-up effects were analyzed using independent t-tests, Mann-Whitney U tests, chi-square for binary variables, or Fisher’s exact test. The paired t-tests or Wilcoxon signed-rank tests were used to analyze treatment, period, and carry-over effects, respectively. Finally, independent t-tests or Mann-Whitney U tests were used to determine differences between groups. The level of statistical significance for all tests was set at P < 0.05, as shown in Fig 2.

## Results

### Demographic characteristics of subjects

The demographic data of the subjects are shown in Table 1. Only subjects with GMFCS levels II and III were able to complete the EVGS in this study. No significant differences were observed in the demographic characteristics of children with CP between the two groups.

**Table 1:** Demographic characteristics of {all subjects} and [subjects of EVGS]#.

Characteristics	Group 1	Group 2	t/χ2	P-Value
	{n = 11}[n = 9]	{n = 12}[n = 9]		
Male	{6}	{5}		{0.684} * Ψ
	[5]	[4]		[>0.05] * Ψ
Age (years)	{5.56±1.97}	{5.25±2.01}	{0.356}	{0.725} Ω
	[5.33±2.00]	[5.22±1.99]	[0.118]	[0.907] Ω
Height (cm)	{110.73±12.37}	{105.17±15.29}	{0.953}	{0.351} Ω
	[110.33±12.95]	[104.89±14.97]	[0.825]	[0.421] Ω
Weight (kg)	{20.41±4.52}	{20.12±5.01}	{0.146}	{0.885} Ω
	[19.98±4.75]	[20.16±4.95]	[-0.078]	[0.939] Ψ
GMFCS level, n				
II	{[5]}	{[5]}		
III	{[4]}	{[4]}	[0.157]	[0.925] Ψ
IV	[2]	[3]		
Tone abnormality				
Spastic	{4}[3]	{4}[3]		
Dyskinetic	{4}[3]	{4}[3]	[0.100]	[0.951]
Mixed	{3}[3]	{4}[3]		

#: {} indicates the statistical data of all objects; [] indicates the statistical data of subjects of EVGS

*: Fisher’s exact test

Ψ: P-value derived from χ the Chi square test

Ω: P-value derived from the paired t test.

### Training parameter of RAGT device

There was no difference in the parameter of the RAGT device between the two groups, as shown in Table 2.

**Table 2:** RAGT device training parameter of {all subjects} and [subjects of EVGS]#.

	Group 1	Group 2	t	P-Value
Gaint cycle (seconds)	{3.60±0.91}	{3.78±0.99}	{-0.447}	{0.659}
	[3.27±0.58]	[3.32±0.63]	[-0.194]	[0.848]

### Safety indicators

#### Adverse events

Three subjects experienced skin pain during the first exercise of the trial phase, and two subjects experienced joint pain during the first exercise of the trial phase. Two subjects developed joint pain and skin pain, respectively, during the second exercise in the trial phase. Two other subjects developed skin lesions on the fifth day of the third week and the first day of the fourth week, respectively. No other types of adverse events have occurred.

#### The changes in heart rate and blood pressure

**Fig 3:**
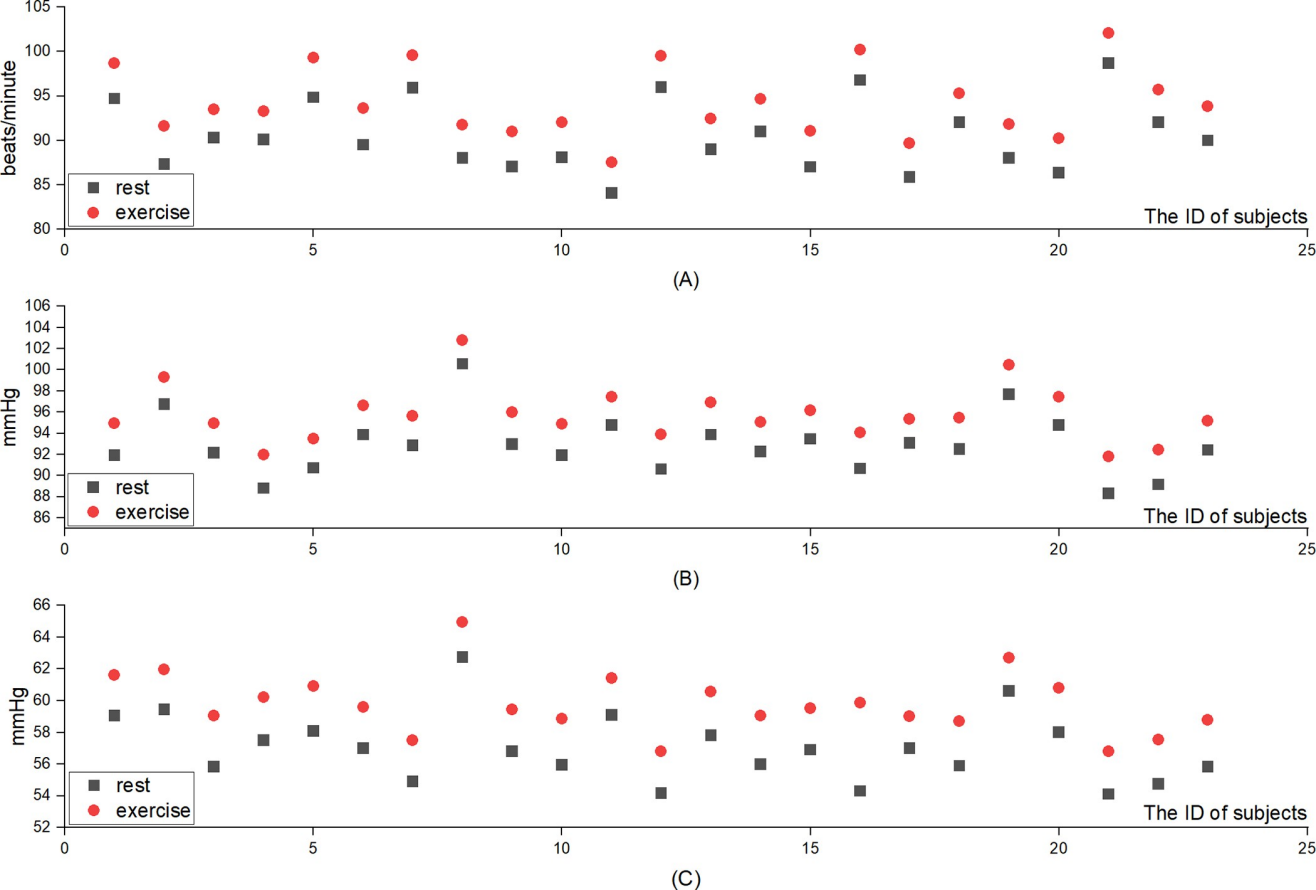
(A) Heart rate; (B) Systolic blood pressure; (C) Diastolic blood pressure.

23 subjects each performed 20 sessions of AiWalker-K training. The results showed that the average heart rate, average systolic blood pressure, and average diastolic blood pressure of each subject during 20 exercise sessions were significantly higher than those at rest (P < 0.05) (S1 File). However, heart rate and blood pressure during training remained within the normal range, as shown in Fig 3. More detailed data on heart rate and blood pressure are provided in the S1 File.

### Effectivity indicators

#### Baseline functional assessment comparison

There were no significant differences in the baseline functional assessments between the two groups, as shown in Table 3.

**Table 3:** Baseline functional characteristics of the two groups.

	Group 1	Group 2	t	P-Value
6MWT #	121.27±33.19	109.00±32.86	0.890	0.383
GMFM-88 (D)	14.00±9.76	13.17±7.42	0.232	0.819
GMFM-88 (E)	11.36±9.35	10.25±6.47	0.335	0.741
PBS	21.82±11.85	21.08±11.08	0.154	0.879
PCI	0.67±0.21	0.77±0.26	-0.973	0.341
EVGS*	19.33±7.30	20.00±5.34	-0.221	0.828

*: Subjects with GMFCS level II and III; #: the main effectivity indicator.

#### Efficacy evaluation comparison

**Fig 4:**
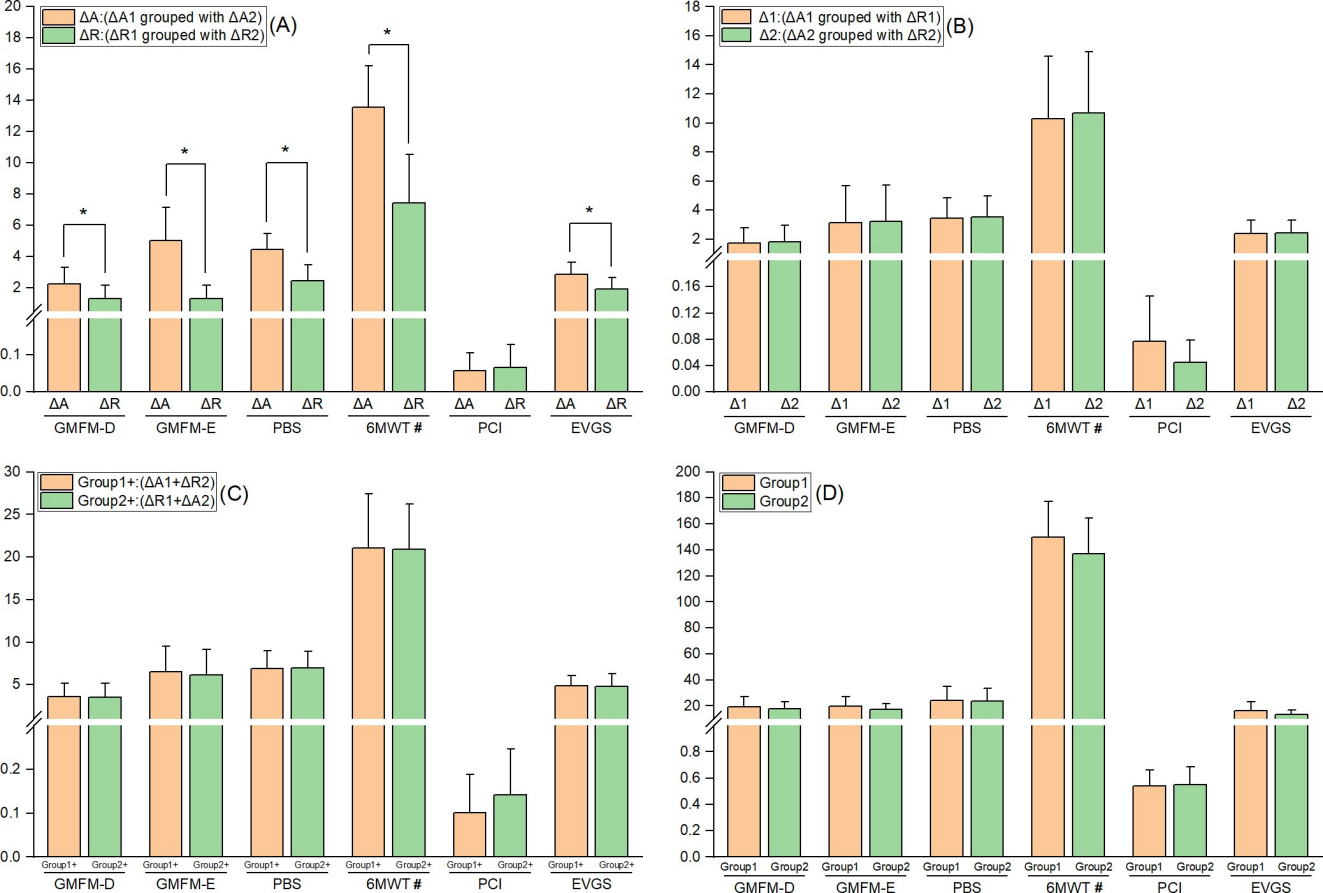
Efficacy evaluation comparison. (A) Treatment effects; (B) Period effects; (C) Carry-over effects; (D) Follow-up effects. *: P < 0.05; #: the main effectivity indicator.

The combination treatment demonstrated superior therapeutic effects on the 6MWT, GMFM-88 (D) and (E) areas, PBS, and EVGS. However, in terms of PCI, there was no significant difference between the combination treatment and routine treatment. None of the period, carry-over, and follow-up effects showed statistical significance, as shown in Fig 4. More detailed data are provided in the S2 File.

## Discussion

The AiWalker-K is a RAGT device designed specifically for rehabilitating children with lower limbs exercise dysfunction. The results of this study indicate that AiWalker-K is safe, but caution should be taken as it may cause skin, joint pain, and even skin damage in children with CP. When combined with routine rehabilitation treatment, AiWalker-K outperforms routine rehabilitation treatment in 6MWT, GMFM-88 (D and E), PBS, and EVGS. The goal is to provide a new treatment option for the rehabilitation of lower limbs in children with CP.

This study primarily tested three safety indicators. First, the occurrence of adverse events; three types of adverse events took place during 23 × 20 experiments. The first type of adverse event was joint pain, which occurred in the early stage of the experiment. It was mainly caused by the knee joints being loosened due to the fixation of the thigh and lower limbs restraint belt of the RAGT device when the device was exercised for about 10 minutes. This resulted in the knee joints being out of their original corresponding positions and causing twisted pain. The researchers subsequently added a restraint belt to the subject’s thigh, and this adverse event did not occur in subsequent experiments. The second and third types of adverse events were skin pain and skin damage, respectively. Skin pain occurred in the early stage of the experiment, and skin damage occurred in the later stage. The reason for this was that the subject’s clothing rubbed against the restraint belt of the lower limbs, causing skin pain. Although the painful episodes did not recur after increasing the application of medical bandages at the friction point in the early stage, there were still two cases of minor skin lesions in the later period. The researchers did not perform a detailed examination of the children’s lower limbs at the beginning and end of each experiment, and studies have indicated that skin color must be checked very carefully before and after each use of a RAGT device, as skin damage from friction may be painless [27]. Other safety indicators were the changes in heart rate and blood pressure. The AiWalker-K is a passive RAGT device, and the subject doesn’t need to exert force actively during rehabilitation exercises. This study shows that the heart rate, systolic blood pressure, and diastolic blood pressure of the subjects during exercises are higher than those at rest, and there are differences. However, the relevant indicators during exercise are still within the acceptable range for children [28, 29]. Although some adverse events occurred in the early stage of the experiment, when the researchers used the AiWalker-K with increasing frequency and experience, almost no adverse events occurred in subsequent experiments. These results demonstrate that under the guidance of experienced personnel, the application of AiWalker-K in the rehabilitation of the lower limbs of children with CP is safe.

Feasibility was evaluated from the following perspectives. First, the motor function was measured, and the rehabilitation effect of iWalker-K combined with routine rehabilitation treatment in GMFM-88 (D) and GMFM-88 (E) areas was found to be better than that of routine rehabilitation treatment, with a statistically significant difference. Previous studies have also shown that children with CP have exhibited significantly improved scores in these areas after RAGT exercises [30–32]. In this study, the mean score change in the GMFM-88 (D) area was 2.26, and a recent study suggested a mean GMFM-88 (D) area score change of 1.2 as the minimal clinically important difference (MCID) [33]. The same study indicated that the MCID of the GMFM-88 (E) area is 0.3 and only GMFCS Level I-IV participants are involved [32]. Another recent study demonstrated that the MCID of the GMFM-88 (E) area in GMFCS Level I-III participants was 1.2 [34]. However, the mean score change in the GMFM-88 (E) area treated with combination treatment in this study was 5.04. Secondly, in balance testing, AiWalker-K combined with routine rehabilitation treatment proved to be superior to routine rehabilitation treatment training. A recent systematic review supported the use of RAGT to optimize knee and hip extension during the standing phase, reduce metabolic costs of gait in children with CP, and increase the activity of leg flexors and extensors [35]. The RAGT device can also promote the activation of trunk muscles to ensure that the body maintains proper alignment and improves static and dynamic equilibrium during the center of gravity transfer movement [36, 37]. Thirdly, compared to routine rehabilitation treatment, AiWalker-K combined with routine rehabilitation treatment significantly improved the 6MWT of children with CP. The latest research shows that combining RAGT and routine rehabilitation treatment can significantly improve the 6MWT test scores of children with CP compared to routine rehabilitation treatment alone [38], which is consistent with the results of this study. However, some studies on RAGT have also indicated that the combination of RAGT and routine rehabilitation treatment does not have an advantage in improving the performance of 6MWT compared to routine rehabilitation treatment alone [39]. Nonetheless, the types of RAGT devices used in the 6MWT tests, including those in this study, vary. It is worth investigating whether the results of the 6MWT test are related to the type of RAGT device or other factors. The reason may be related to factors such as device type (mobile/treadmill), support type (active/passive), and weight support. Furthermore, PCI is a commonly used indicator for measuring walking energy expenditure, which can be obtained through simple heart rate changes and walking speed [19]. In this study, there was no statistically significant difference in PCI indicators between AiWalker-K combined with routine rehabilitation treatment and routine rehabilitation treatment. This may be because AiWalker K is a passive training device, and children with CP do not need to exercise force activity. Therefore, there is no impact on energy consumption. AiWalker-K combined with routine rehabilitation treatment also significantly improved gait and posture scores in children with CP compared to routine rehabilitation treatment. Children with CP are more likely to experience biomechanical changes during walking, such as reduced knee flexion during gait swing, excessive knee extension or flexion during gait standing, and other abnormal postures such as hip pronation and excessive hip adduction [40]. The gait posture of RAGT is designed based on the gait posture of normal individuals. Providing repetitive and accurate gait posture training helps to improve the stability and smoothness of gait posture in children with CP [41].

This study was conducted at a single center and utilized a cross-over design to provide a higher sample size with a smaller sample size. However, a washout period was not introduced to avoid the impact of previous treatment on later treatment. Although this study calculated period effects, carry-over effects, and follow-up effects to exclude the impact of the previous intervention on the latter intervention [26], the possibility of additional AiWalker-K training duration affecting the research results cannot be ruled out. Furthermore, patients received both AiWalker-K rehabilitation treatment and routine rehabilitation treatment and did not receive additional treatment during the routine rehabilitation treatment period. In addition, the encouragement of family members may be a confounding factor in the positive effect of RAGT in the study, as motivational feedback has been shown to affect, for example, muscular amplitude [42].

Future research on the AiWalker-K lower limbs rehabilitation device can focus on long-term effects, multi-center studies, comparative studies, studies on specific populations, use experience and user satisfaction studies, and cost-benefit analysis. These studies can further promote the development and application of equipment and provide more basis and guidance for clinical practice.

## Conclusions

AiWalker-K can be safely used during rehabilitation exercises for children with CP, but it should be supervised by experienced medical personnel. When combined with routine rehabilitation treatment, AiWalker-K outperforms routine rehabilitation treatment in multiple efficacy evaluation indicators, such as gross motor function assessment, balance, walking ability, and gait posture. These findings suggest that AiWalker-K can be applied safely for lower limbs rehabilitation exercises in children with CP.

## Supporting information

S1 Checklist CONSORT 2010 checklist of information to include when reporting a randomised trial*. (DOC)

S1 Fig Actual front view of AiWalker-K. (TIF)

S2 Fig Actual side view of AiWalker-K. (TIF)

S3 Fig Actual back view of AiWalker-K. (TIF)

S1 File Heart rate and blood pressure data. (XLSX)

S2 File Efficacy evaluation comparison. (XLSX)

S3 File (DOCX)

## References

[1] Vitrikas K, Dalton H, Breish D. Cerebral Palsy: An Overview. Am Fam Physician. 2020;101: 213–220. Available from: https://www.aafp.org/pubs/afp/issues/2020/0215/p213.html 32053326

[2] Colver A, Fairhurst C, Pharoah POD. Cerebral palsy. Lancet. 2014;383: 1240–1249. doi: 10.1016/S0140-6736(13)61835-8 24268104

[3] Gulati S, Sondhi V. Cerebral Palsy: An Overview. Indian J Pediatr. 2018;85: 1006–1016. doi: 10.1007/s12098-017-2475-1 29152685

[4] Cortés-Pérez I, González-González N, Peinado-Rubia AB, Nieto-Escamez FA, Obrero-Gaitán E, García-López H. Efficacy of Robot-Assisted Gait Therapy Compared to Conventional Therapy or Treadmill Training in Children with Cerebral Palsy: A Systematic Review with Meta-Analysis. Sensors (Basel). 2022;22: 9910. doi: 10.3390/s22249910 36560281 PMC9785795

[5] Wittenberg GF. Neural plasticity and treatment across the lifespan for motor deficits in cerebral palsy. Dev Med Child Neurol. 2009;51 Suppl 4: 130–133. doi: 10.1111/j.1469-8749.2009.03425.x 19740220

[6] Nizamis K, Athanasiou A, Almpani S, Dimitrousis C, Astaras A. Converging Robotic Technologies in Targeted Neural Rehabilitation: A Review of Emerging Solutions and Challenges. Sensors (Basel). 2021;21: 2084. doi: 10.3390/s21062084 33809721 PMC8002299

[7] Kolb B, Harker A, Gibb R. Principles of plasticity in the developing brain. Dev Med Child Neurol. 2017;59: 1218–1223. doi: 10.1111/dmcn.13546 28901550

[8] Lerner ZF, Damiano DL, Bulea TC. The Effects of Exoskeleton Assisted Knee Extension on Lower-Extremity Gait Kinematics, Kinetics, and Muscle Activity in Children with Cerebral Palsy. Sci Rep. 2017;7: 13512. doi: 10.1038/s41598-017-13554-2 29044202 PMC5647342

[9] Drużbicki M, Rusek W, Snela S, Dudek J, Szczepanik M, Zak E, et al. Functional effects of robotic-assisted locomotor treadmill thearapy in children with cerebral palsy. J Rehabil Med. 2013;45: 358–363. doi: 10.2340/16501977-1114 23450428

[10] van Kammen K, Reinders-Messelink HA, Elsinghorst AL, Wesselink CF, Meeuwisse-de Vries B, van der Woude LHV, et al. Amplitude and stride-to-stride variability of muscle activity during Lokomat guided walking and treadmill walking in children with cerebral palsy. Eur J Paediatr Neurol. 2020;29: 108–117. doi: 10.1016/j.ejpn.2020.08.003 32900595

[11] Ouendi N, Hubaut R, Pelayo S, Anceaux F, Wallard L. The rehabilitation robot: factors influencing its use, advantages and limitations in clinical rehabilitation. Disabil Rehabil Assist Technol. 2024;19: 546–557. doi: 10.1080/17483107.2022.2107095 35921160

[12] Calabrò RS, Cacciola A, Bertè F, Manuli A, Leo A, Bramanti A, et al. Robotic gait rehabilitation and substitution devices in neurological disorders: where are we now? Neurol Sci. 2016;37: 503–514. doi: 10.1007/s10072-016-2474-4 26781943

[13] Fundarò C, Giardini A, Maestri R, Traversoni S, Bartolo M, Casale R. Motor and psychosocial impact of robot-assisted gait training in a real-world rehabilitation setting: A pilot study. PLoS One. 2018;13: e0191894. doi: 10.1371/journal.pone.0191894 29444172 PMC5812583

[14] Chen S, Wang Z, Li Y, Tang J, Wang X, Huang L, et al. Safety and Feasibility of a Novel Exoskeleton for Locomotor Rehabilitation of Subjects With Spinal Cord Injury: A Prospective, Multi-Center, and Cross-Over Clinical Trial. Front Neurorobot. 2022;16: 848443. doi: 10.3389/fnbot.2022.848443 35645758 PMC9133609

[15] Schaffarczyk M, Rogers B, Reer R, Gronwald T. Validity of the Polar H10 Sensor for Heart Rate Variability Analysis during Resting State and Incremental Exercise in Recreational Men and Women. Sensors (Basel). 2022;22: 6536. doi: 10.3390/s22176536 36081005 PMC9459793

[16] Maher CA, Williams MT, Olds TS. The six-minute walk test for children with cerebral palsy. Int J Rehabil Res. 2008;31: 185–188. doi: 10.1097/MRR.0b013e32830150f9 18467936

[17] Ko J, Kim M. Reliability and responsiveness of the gross motor function measure-88 in children with cerebral palsy. Phys Ther. 2013;93: 393–400. doi: 10.2522/ptj.20110374 23139425

[18] Franjoine MR, Gunther JS, Taylor MJ. Pediatric balance scale: a modified version of the berg balance scale for the school-age child with mild to moderate motor impairment. Pediatr Phys Ther. 2003;15: 114–128. doi: 10.1097/01.PEP.0000068117.48023.18 17057441

[19] Raja K, Joseph B, Benjamin S, Minocha V, Rana B. Physiological cost index in cerebral palsy: its role in evaluating the efficiency of ambulation. J Pediatr Orthop. 2007;27: 130–136. doi: 10.1097/01.bpb.0000242440.96434.26 17314635

[20] Del Pilar Duque Orozco M, Abousamra O, Church C, Lennon N, Henley J, Rogers KJ, et al. Reliability and validity of Edinburgh visual gait score as an evaluation tool for children with cerebral palsy. Gait Posture. 2016;49: 14–18. doi: 10.1016/j.gaitpost.2016.06.017 27344448

[21] Viehweger E, Pfund LZ, Hélix M, Rohon MA, Jacquemier M, Scavarda D, et al. Influence of clinical and gait analysis experience on reliability of observational gait analysis (Edinburgh Gait Score Reliability). Ann Phys Rehabil Med. 2010 Nov 1;53(9):535–46. doi: 10.1016/j.rehab.2010.09.002 20952267

[22] Aroojis A, Sagade B, Chand S. Usability and Reliability of the Edinburgh Visual Gait Score in Children with Spastic Cerebral Palsy Using Smartphone Slow-Motion Video Technology and a Motion Analysis Application: A Pilot Study. Indian J Orthop. 2021;55: 931–938. doi: 10.1007/s43465-020-00332-y 34194650 PMC8192632

[23] Fernández-González P, Koutsou A, Cuesta-Gómez A, Carratalá-Tejada M, Miangolarra-Page JC, Molina-Rueda F. Reliability of Kinovea® Software and Agreement with a Three-Dimensional Motion System for Gait Analysis in Healthy Subjects. Sensors (Basel). 2020;20: 3154. doi: 10.3390/s20113154 32498380 PMC7308968

[24] Siyasinghe NM, Sooriyarachchi MR. Guidelines for calculating sample size in 2x2 crossover trials: a simulation study. 2011;39: 77. doi: 10.4038/jnsfsr.v39i1.2929

[25] McCormick AM, Alazem H, Zaidi S, Barrowman NJ, Ward LM, McMillan HJ, et al. A randomized, cross-over trial comparing the effect of innovative robotic gait training and functional clinical therapy in children with cerebral palsy; a protocol to test feasibility. Contemp Clin Trials. 2023;127: 107086. doi: 10.1016/j.cct.2023.107086 36669727

[26] Kenward MG, Jones B. 15 Design and Analysis of Cross-Over Trials. Handbook of Statistics. Elsevier; 2007 Jan 1;27:464–490. doi: 10.1016/S0169-7161(07)27015-4

[27] Kolakowsky-Hayner SA, Crew J, Moran S, Shah A. Safety and Feasibility of using the EksoTM Bionic Exoskeleton to Aid Ambulation after Spinal Cord Injury. J Spine S4: 003. doi: 10.4172/2165-7939.s4-003

[28] Cossio-Bolaños M, Vidal-Espinoza R, de Campos FCC, Sulla-Torres J, Cossio-Bolaños W, Andruske CL, et al. Establishing percentiles for blood pressure based on absolute height for children and adolescents. BMC Pediatr. 2021;21: 26. doi: 10.1186/s12887-020-02489-9 33413191 PMC7792128

[29] Kholod H, Jamil A, Katz-Leurer M. The associations between motor ability, walking activity and heart rate and heart rate variability parameters among children with cerebral palsy and typically developed controls. NeuroRehabilitation. 2013;33: 113–119. doi: 10.3233/NRE-130934 23949027

[30] Schroeder AS, Homburg M, Warken B, Auffermann H, Koerte I, Berweck S, et al. Prospective controlled cohort study to evaluate changes of function, activity and participation in patients with bilateral spastic cerebral palsy after Robot-enhanced repetitive treadmill therapy. Eur J Paediatr Neurol. 2014;18: 502–510. doi: 10.1016/j.ejpn.2014.04.012 24821475

[31] Wallard L, Dietrich G, Kerlirzin Y, Bredin J. Robotic-assisted gait training improves walking abilities in diplegic children with cerebral palsy. Eur J Paediatr Neurol. 2017;21: 557–564. doi: 10.1016/j.ejpn.2017.01.012 28188024

[32] Wallard L, Dietrich G, Kerlirzin Y, Bredin J. Effect of robotic-assisted gait rehabilitation on dynamic equilibrium control in the gait of children with cerebral palsy. Gait Posture. 2018;60: 55–60. doi: 10.1016/j.gaitpost.2017.11.007 29156378

[33] Storm FA, Petrarca M, Beretta E, Strazzer S, Piccinini L, Maghini C, et al. Minimum Clinically Important Difference of Gross Motor Function and Gait Endurance in Children with Motor Impairment: A Comparison of Distribution-Based Approaches. Biomed Res Int. 2020;2020: 2794036. doi: 10.1155/2020/2794036 32509855 PMC7246400

[34] Oeffinger D, Bagley A, Rogers S, Gorton G, Kryscio R, Abel M, et al. Outcome tools used for ambulatory children with cerebral palsy: responsiveness and minimum clinically important differences. Dev Med Child Neurol. 2008;50: 918–925. doi: 10.1111/j.1469-8749.2008.03150.x 19046185 PMC2990955

[35] Hunt M, Everaert L, Brown M, Muraru L, Hatzidimitriadou E, Desloovere K. Effectiveness of robotic exoskeletons for improving gait in children with cerebral palsy: A systematic review. Gait Posture. 2022;98: 343–354. doi: 10.1016/j.gaitpost.2022.09.082 36306544

[36] Hong J, Lee J, Choi T, Choi W, Kim T, Kwak K, et al. Feasibility of Overground Gait Training Using a Joint-Torque-Assisting Wearable Exoskeletal Robot in Children with Static Brain Injury. Sensors (Basel). 2022;22: 3870. doi: 10.3390/s22103870 35632279 PMC9144762

[37] Žarković D, Šorfová M, Tufano JJ, Kutílek P, Vítečková S, Ravnik D, et al. Gait changes following robot-assisted gait training in children with cerebral palsy. Physiol Res. 2021;70: S397–S408. doi: 10.33549/physiolres.934840 35099258 PMC8884401

[38] Fu W-S, Song Y-C, Wu B-A, Qu C-H, Zhao J-F. Virtual reality combined with robot-assisted gait training to improve walking ability of children with cerebral palsy: A randomized controlled trial. Technol Health Care. 2022;30: 1525–1533. doi: 10.3233/THC-212821 35661029

[39] Moll F, Kessel A, Bonetto A, Stresow J, Herten M, Dudda M, et al. Use of Robot-Assisted Gait Training in Pediatric Patients with Cerebral Palsy in an Inpatient Setting-A Randomized Controlled Trial. Sensors (Basel). 2022;22: 9946. doi: 10.3390/s22249946 36560316 PMC9783925

[40] Rethlefsen SA, Blumstein G, Kay RM, Dorey F, Wren TAL. Prevalence of specific gait abnormalities in children with cerebral palsy revisited: influence of age, prior surgery, and Gross Motor Function Classification System level. Dev Med Child Neurol. 2017;59: 79–88. doi: 10.1111/dmcn.13205 27421715

[41] Bonanno M, Militi A, La Fauci Belponer F, De Luca R, Leonetti D, Quartarone A, et al. Rehabilitation of Gait and Balance in Cerebral Palsy: A Scoping Review on the Use of Robotics with Biomechanical Implications. J Clin Med. 2023;12: 3278. doi: 10.3390/jcm12093278 37176718 PMC10179520

[42] Aurich Schuler T, Müller R, van Hedel HJ. Leg surface electromyography patterns in children with neuro-orthopedic disorders walking on a treadmill unassisted and assisted by a robot with and without encouragement. J Neuroeng Rehabil. 2013;10: 78. doi: 10.1186/1743-0003-10-78 23867005 PMC3720176

