# Supplementary figures and images for: Clinical study on the safety and feasibility of AiWalker-K for lower limbs exercise rehabilitation in children with cerebral palsy
Source: PLoS One. 2024 May 22;19(5):e0303517. doi: 10.1371/journal.pone.0303517 (PMC11111022; doi:10.1371/journal.pone.0303517)

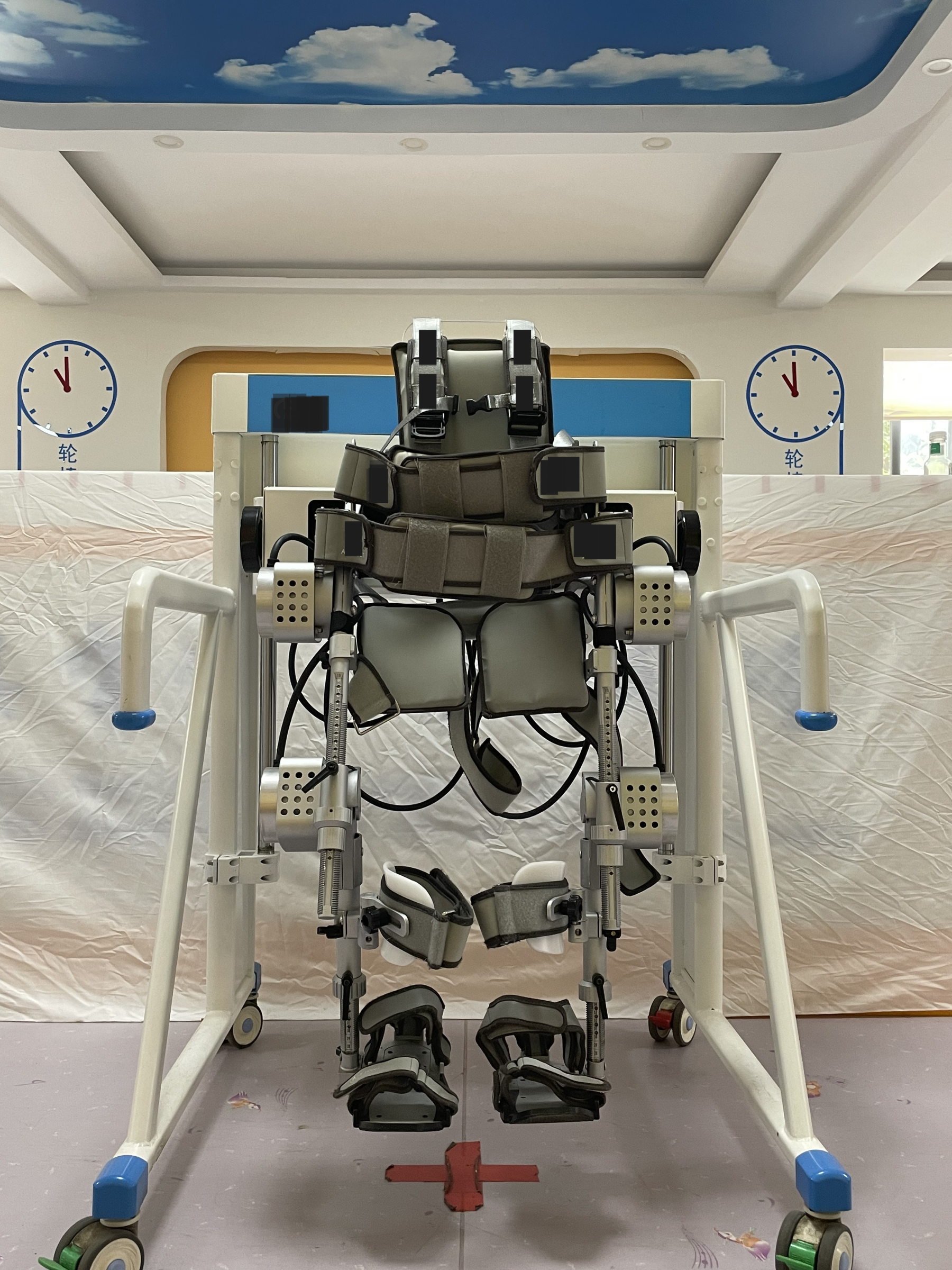

Supplement: S1 Fig — (TIF) [file pone.0303517.s002.tif]

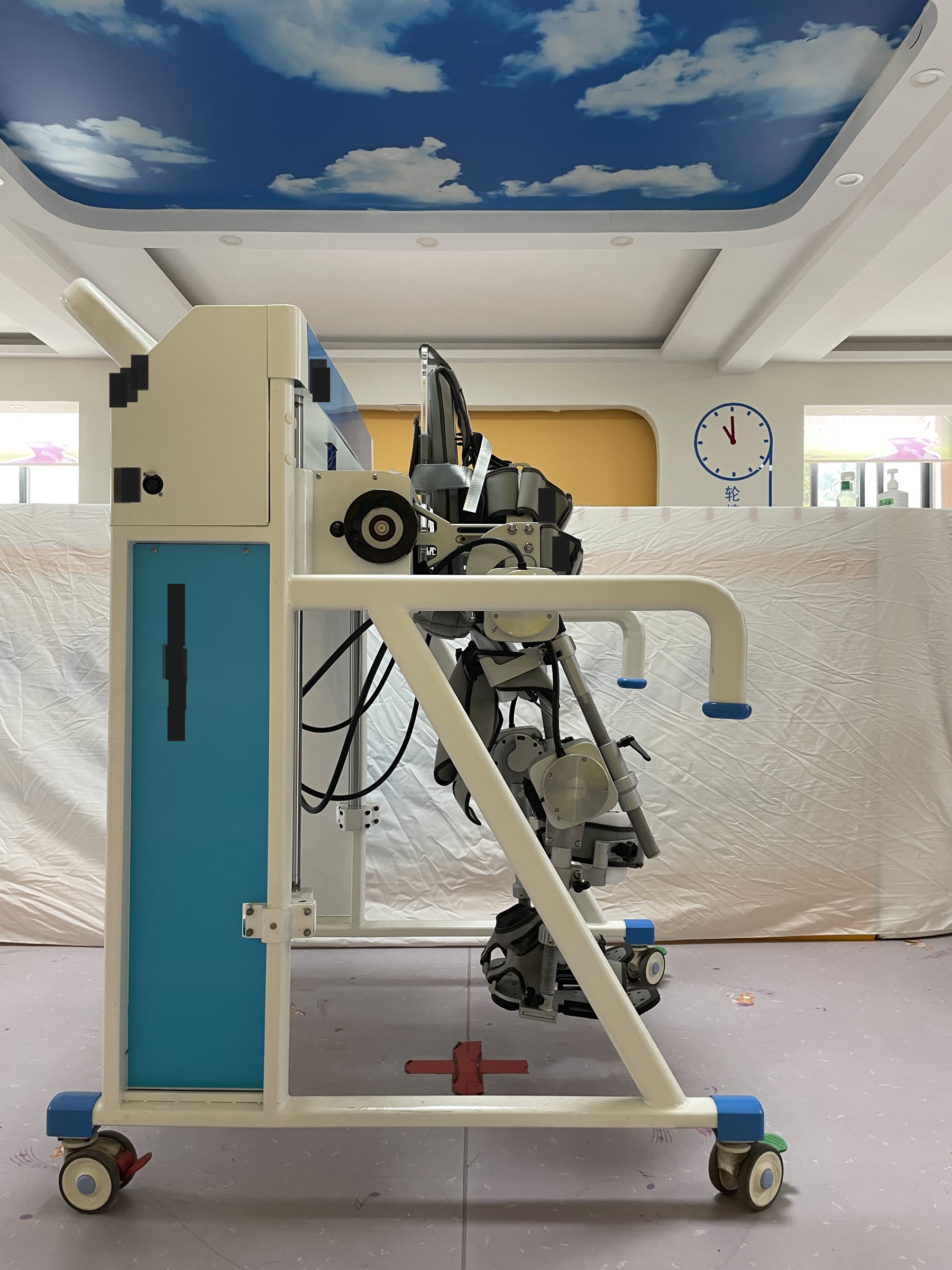

Supplement: S2 Fig — (TIF) [file pone.0303517.s003.tif]

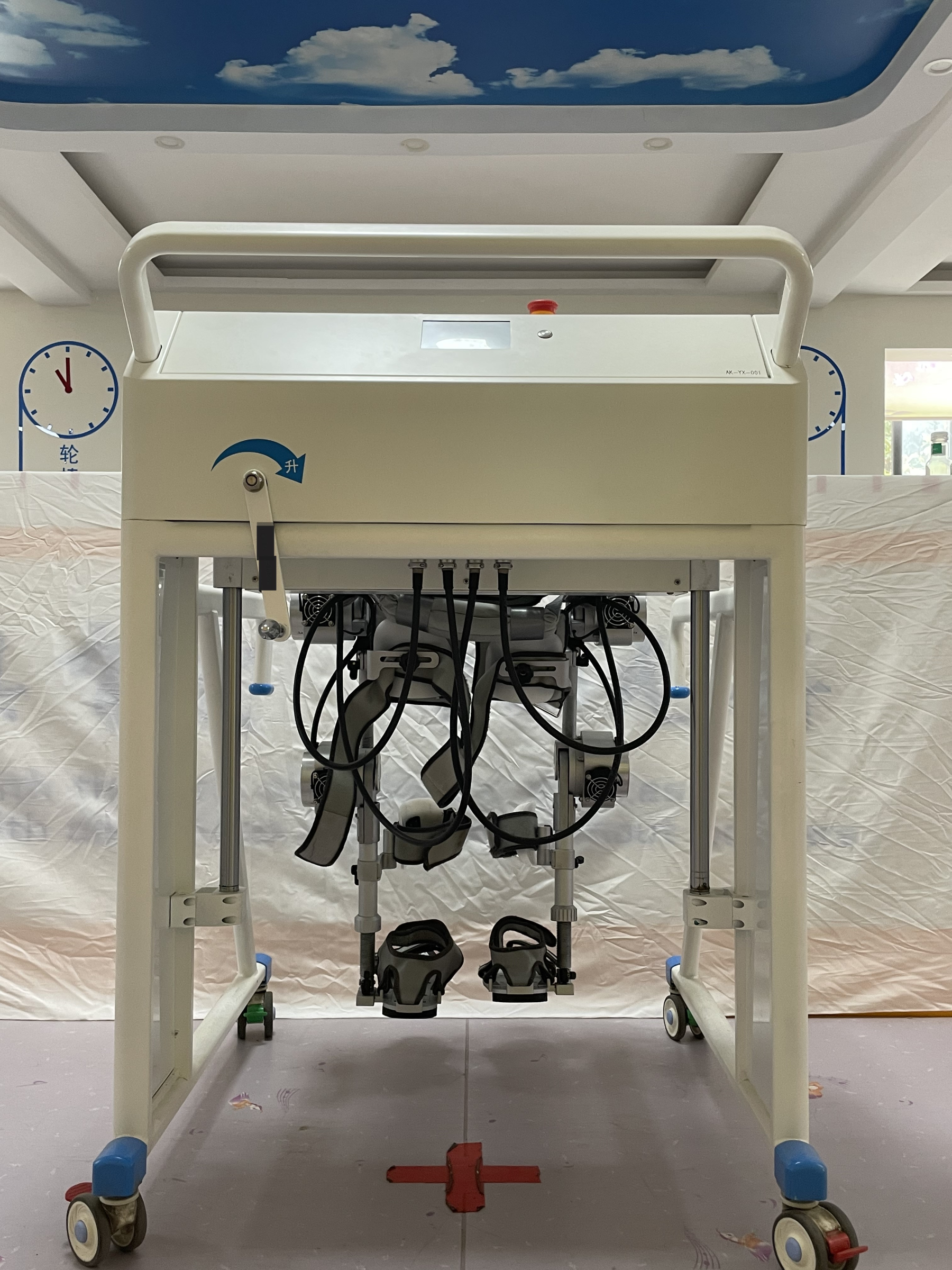

Supplement: S3 Fig — (TIF) [file pone.0303517.s004.tif]
