# Supplementary material for: Clinical study on the safety and feasibility of AiWalker-K for lower limbs exercise rehabilitation in children with cerebral palsy
Source: PLoS One. 2024 May 22;19(5):e0303517. doi: 10.1371/journal.pone.0303517 (PMC11111022; doi:10.1371/journal.pone.0303517)
Supplement: S3 File — (DOCX) [file pone.0303517.s007.docx]

**（Clinical study on the safety and feasibility of AiWalker-K for lower limbs exercise rehabilitation in children with cerebral palsy）study protocol**

**Version number and date：（third edition 2020-8-31）**

**Experimental Purpose and Background**

The incidence of cerebral palsy is approximately 2.11 cases per 1000 live births. It is a permanent group of central motor and postural developmental disorders, characterized by a syndrome of restricted activity caused by non-progressive damage to the brain during development. It is often accompanied by sensory, perceptual, cognitive, communication, behavioral, and other impairments, as well as seizures. Its main manifestations are motor disorders and postural abnormalities [^[[1]](#endnote-1)^-^[[2]](#endnote-2)^].Many children with severe cerebral palsy can only rely on assistive devices to walk or even sit in wheelchairs for life, which imposes a significant burden on both society and families.

The emergence of exoskeleton robot technology can benefit children with cerebral palsy in three main aspects: (1) It can facilitate large-scale repetitive walking, helping them improve and restore their walking ability. (2) It reduces the need for intensive physical labor by medical professionals, making extensive walking training feasible and potentially shortening the rehabilitation duration. (3) It helps reduce complications such as pain, spasms, osteoporosis, while also improving cardiovascular, lower urinary, and intestinal functions.

For children with cerebral palsy who have limited upper limb and trunk function, low endurance, or cognitive impairment, adequate trunk fixation and support are essential during walking. However, existing stationary gait robots often have large dimensions, requiring children to be transferred to specialized treatment rooms for equipment usage, which restricts their ability to use the equipment outdoors. Moreover, the high cost of these devices results in a scarcity of hospitals equipped with such technology, leading to a low adoption rate.

Currently, there are only a limited number of exoskeleton robots available that can effectively address these challenges. In response to this need, Beijing Daai Robot Technology Co., Ltd. has developed the AiWalker-K lower limb motion rehabilitation robot (Figure 1). The AiWalker-K exhibits several distinctive features, including its compact size, easy mobility, and relatively affordable price, making it convenient to be transported directly to any ward or even bedside. It may also be suitable for home use. Subjects can effortlessly transition from a wheelchair to the AiWalker-K, securing themselves with the provided straps, and adjusting to a standing position. The walking exercises can be performed in mid-air or on actual ground, with the flexibility to modify speed and stride as required. By perceiving the external environment in motion and undergoing transformation, subjects experience enriched visual stimulation, which enhances their engagement in the treatment process and contributes to improved emotional well-being. The compact design of the AiWalker-K enables a wide range of clinical applications, accommodating children with quadriplegia, cognitive impairments, ataxia, and even those confined to the ward. Particularly, it holds great potential for long-term home rehabilitation for children with cerebral palsy, aiding in the reduction of complications.

Building upon the aforementioned advantages of the AiWalker-K, the objective of this clinical study is to assess the device's safety, walking efficiency, physiological impact, and its potential to enhance gait in children with cerebral palsy undergoing lower limb motor rehabilitation.

**Experimental route and ethical issues involved**

**1. Research object:** Children with cerebral palsy.

**2. Diagnostic criteria:** Diagnosis and classification criteria for cerebral palsy in the definition, diagnostic criteria, and clinical classification of cerebral palsy (2022): **Diagnosis:** ①Persistent central motor dysfunction; ②Abnormal development of movement and posture; ③Abnormal muscle tone and strength; ④Abnormal reflex development. **Clinical classification:** ①spastic quadriplegia; ②Spastic diplegia; ③Spastic hemiplegia; ④Non random movement type; ⑤Ataxia type; ⑥Hybrid.

**3. Inclusion criteria:** ①Conforming to the diagnostic and classification criteria for cerebral palsy and with GMFCS level II-IV; ②Age range from 3 to 10 years old; ③The required height of the equipment is 80cm~150cm, and the weight is ≤ 70kg; ④Be able to reliably report pain, fear, and discomfort, as well as understand instructions and cooperate in completing training; ⑤Those who have not undergone surgical correction surgery or botulinum toxin injection treatment in the past 6 months; ⑥If taking anti spasmodic drugs orally, their medication status must be stable for at least one month before enrollment in the study; ⑦Fully understand the content of the experiment, voluntarily participate in the experiment, and the subject's guardian signs an informed consent form in writing. When the subject has the ability to give consent, the subject also needs to sign an informed consent form in person.

**4. Exclusion criteria:** ①Severe lower limb contracture; ②Complications with important organs such as heart, lung, liver, and kidney; ③Accompanied by severe epilepsy, genetic metabolic diseases, and severe skeletal system diseases; ④Merge visual, auditory, and intellectual disabilities; ⑤Concomitant acute or progressive neurological diseases; ⑥Skin damage or infection in contact with equipment; ⑦Inflammation in the lower limbs and limitation of joint activity; ⑧This study participated in clinical trials of other lower limb rehabilitation within one month before randomization; ⑨Children who plan or anticipate using new drugs, surgeries, or other anti spastic treatments during this study period The researchers believe that there are other factors that are not suitable for children to participate in this study.

**5. Dropout criteria:** ①Termination of treatment due to various reasons such as adverse events; ②Violation of treatment plan due to poor compliance; ③Those who undergo surgical correction surgery or botulinum toxin injection during treatment.

**6. Research methods:**

Because this is the first clinical application of AiWalker K in lower limb rehabilitation research for children with cerebral palsy, the study is divided into preparation stage, experimental stage, and data recording stage. When using the device, the child requires the therapist to control the direction of walking behind the device and ensure the safety of the child during the device use phase. All participants' relatives were informed in detail of the purpose of the study and the benefits and risks of participating in the study.

6.1 Preparation stage

The child overcomes their fear of equipment with the help of relatives and therapists. Before using the equipment, first check whether the equipment is abnormal under different gait cycles, measure the size of the child's relevant body parts (pelvic width and leg length), the initial angle of bilateral hip and knee joints, and the range of motion of bilateral hip and knee joints, and then adjust the length of bilateral leg rods, the initial angle of bilateral hip and knee joints, and the range of motion of bilateral hip and knee joints, And ensure that the initial angle and rotation center of the equipment's bilateral hip and knee joint motors correspond to the initial angle and rotation center of the child's bilateral hip and knee joints, and the activity of the equipment's bilateral hip and knee joint motors is consistent with the corresponding activity of the child's bilateral hip and knee joints, and record the above information in a record. After wearing, adjust the equipment to make the bottom of the child's foot off the ground in a state of full weight loss. Adjust the gait cycle to 5.25 seconds at most and turn on the equipment to make the child's lower limbs move passively under the drive of the equipment. Gradually adjust the gait cycle to the best. The choice of the best gait cycle should be formulated by the rehabilitation therapist according to the child's own conditions and the child's tolerance. During the period, observe whether the child has any discomfort, If discomfort occurs, press the emergency stop button in a timely manner and take corresponding measures for the child, recording the optimal gait cycle. Adjust the equipment to allow the child to walk on the test site according to the experimental method, increase the feedback of the child's plantar mechanics, and promptly handle any discomfort during this period. Each child is given a maximum of three pull-through and walking tests. Please refer to Attachment 1 for equipment operation instructions.

6.2 Test phase

This is a single center, single blind, randomized crossover trial. AiWalker K training (A) and routine rehabilitation training (R) were both required to be applied to children in the experimental stage. According to the random grouping method, the children were evenly divided into AR group and RAR group. Baseline evaluation was conducted on all patients before the trial. The AR group consists of two stages. The first stage is AiWalker K training combined with routine rehabilitation training and is evaluated after this stage. The second stage is routine rehabilitation training and is evaluated after this stage of training; The RAR group consists of three stages. The first stage is routine rehabilitation training and is evaluated after this stage. The second stage is AiWalker K training combined with routine rehabilitation training and is evaluated after this stage of training. The third stage is still routine rehabilitation training and is evaluated after this stage of training. All stages are 4 weeks. The research plan and timeline are shown in the technical roadmap. During the entire rehabilitation treatment process, the therapist should pay attention to observing the condition of the child to ensure their safety. In case of emergency, training should be suspended, and all trials should be completed within 6 months.

AiWalker K training is conducted 5 times a week for 4 weeks, with a duration of 30 minutes per session, excluding the time for putting on and off equipment. Children are required to remain quiet 10 minutes before the training. The gait cycle should be the most suitable gait cycle selected during the preparation phase and cannot be changed. Rehabilitation therapists should not impose non emergency interference on the equipment while ensuring safety behind it, only ensuring the direction of equipment travel and the safety of the child. Family members can provide encouragement and support to the child next to the trail.

All children will continue to receive routine rehabilitation treatment plans for themselves during the experimental phase, including exercise therapy, paraffin therapy, neuromuscular electrical stimulation therapy, and massage therapy. Exercise therapy: including muscle strength exercises, activity exercises, balance exercises, etc., 30 minutes per session, 2 times a day, 10 times a week; Paraffin therapy: Using the wax cake method at a temperature of 52 ℃ to 55 ℃, apply it to the corresponding anatomical location of the spasmodic muscle once a day, 30 minutes each time, and 5 times a week; Neuromuscular electrical stimulation therapy: Using the bipolar method, find the motor points to be stimulated and stimulate the corresponding muscles, with a frequency of 1Hz and a pulse width of 100ms, once a day for 30 minutes each time, 5 times a week; Massage therapy: Operated by a uniformly trained massage therapist, once a day, five times a week, for 30 minutes each time. If there are no special circumstances, the patient will not change the type and frequency of intervention during the study period.

6.3 Data recording stage

Before the start of the experiment, record the general information of the patient, such as age, gender, height, weight, GMFCS level, and clinical classification of cerebral palsy. All patients recorded adverse events, percentage increase in average heart rate, and changes in blood pressure during AiWalker K training. During the evaluation stage, the Gross Motor Function Test Scale (C-zone, D-zone, and E-zone), Child Balance Scale, 6-minute walking distance test, Physical Consumption Index, and Edinburgh Visual Gait Scale were all evaluated. If the child is unable to undergo an assessment due to physical reasons, the assessment will be invalidated and recorded.

**7. Observation indicators and efficacy evaluation**

7.1 Safety indicators

(1) Adverse events: pain, falls, skin injuries, joint injuries, fractures, etc

(2) Average percentage increase in heart rate (% HRI): Electrocardiograms during the "HRwalk" and "HRbefore" periods were recorded using the Polar H10 heart rate chest band The HRI calculation formula is as follows:

$$\%HRI=\frac{HRwalk-HRbefore}{HRbefore}\times100$$

Among them, "HRwalk" refers to the average heart rate determined by the Polar H10 heart rate chest band during the training period (stable period) between 10min and 20min, and "HRbefore" refers to the average heart rate determined by the Polar H10 heart rate chest band 1 minute before the start of the experiment after sufficient rest to calculate the average heart rate under quiet state.

(3) Blood pressure changes: Use a calibrated medical electronic blood pressure monitor to measure blood pressure twice. The first measurement of upper limb blood pressure was taken in a sitting position before the start of the walking test. The second blood pressure measurement was taken within 1 minute after the end of the walking test from the same position on the same upper limb. The child should still be in the device during the second blood pressure measurement, and the device can only be removed after the measurement is completed.

7.2 Feasibility indicators

(1) Gross motor function measure (GMFM-88): mainly used to measure the changes in gross motor function over time or due to intervention in children with cerebral palsy. It is currently the most widely used scale in the assessment of gross motor function in children with cerebral palsy. There are a total of 88 evaluation items in the scale, each of which adopts a 4-level scoring method and is divided into five functional areas: Area A lying and rolling (17 items), Area B sitting (20 items), Area C climbing and kneeling (14 items), Area D station (13 items), Area E walking, running and jumping (24 items). GMFM88 items belong to the sequential scale, and the five functional areas can be evaluated alone or in combination. In this experiment, the C, D, and E functional areas were selected as observation indicators, and the evaluation results include C, D, and E The original fraction and percentage of the E energy region.

(2) Pediatric Balance Scale (PBS): PBS is a revised version of the Berg, s Balance Scale (BBS), used to test the balance ability of children with mild to moderate motor disorders. The scale consists of 14 items, ranging from 0 (minimum function) to 4 (maximum function), with a maximum score of 56, and has good reliability. PBS only evaluates subjects who can stand alone for a period of time.

(3) 6 Minutes Walking Test (6MWT): 6MWT is commonly used to test gait endurance in children with cerebral palsy. The child walks for 6 minutes at the most suitable speed of their choice on a 50 meter round-trip trail. When they feel unable to continue, they can rest, record the total walking distance, and use auxiliary equipment. Each participant should use the same assistive equipment for all evaluations.

(4) Physical cost index (PCI): It is a commonly used indicator for measuring walking energy consumption, which is simple and easy to implement. The heart rate testing tool is the Polar H10 heart rate chest strap. Firstly, measure the patient's resting heart rate (RestHR). Then measure the stable heart rate (WorkingHR) of the child while using AiWalker K, and the duration of stable heart rate must be greater than 4 minutes. Measure the walking distance of the child using AiWalker K within 30 minutes and obtain an average speed of V (m/min). The calculation formula for PCI (beats/m) is as follows:

$$PCI=\frac{WorkingHR-RestHR}{V}$$

Among them, the higher the PCI value, the higher the energy consumption per unit time of walking.

(5) Edinburgh Visual Gait Score (EVGS): EVGS is an effective and reliable clinical evaluation tool for visual gait analysis in children with cerebral palsy. The child walks at an optimal speed on an 8-meter-long trail with or without the use of assistive devices(Figure 2). Place a high-speed camera at one end and in the middle of the trail to capture the walking video of the child, as shown in the schematic diagram below. The walking data was processed and analyzed using Kinovea motion video analysis software for two to three gait cycles during the stable walking stage of the child. This software has good credibility in motion video analysis.


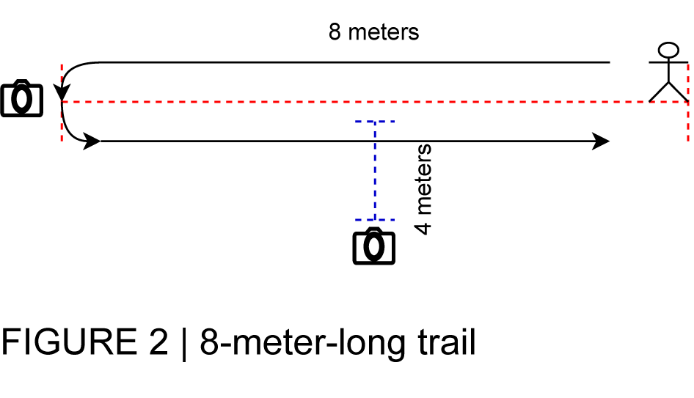


Figure 2

7.3 Efficacy evaluation:

Three evaluators with more than 5 years of work experience were selected to conduct the evaluations. The mean value of their assessments was used, and the evaluators were not informed of the interventions and sequence of each subject.

**8. Technical roadmap**


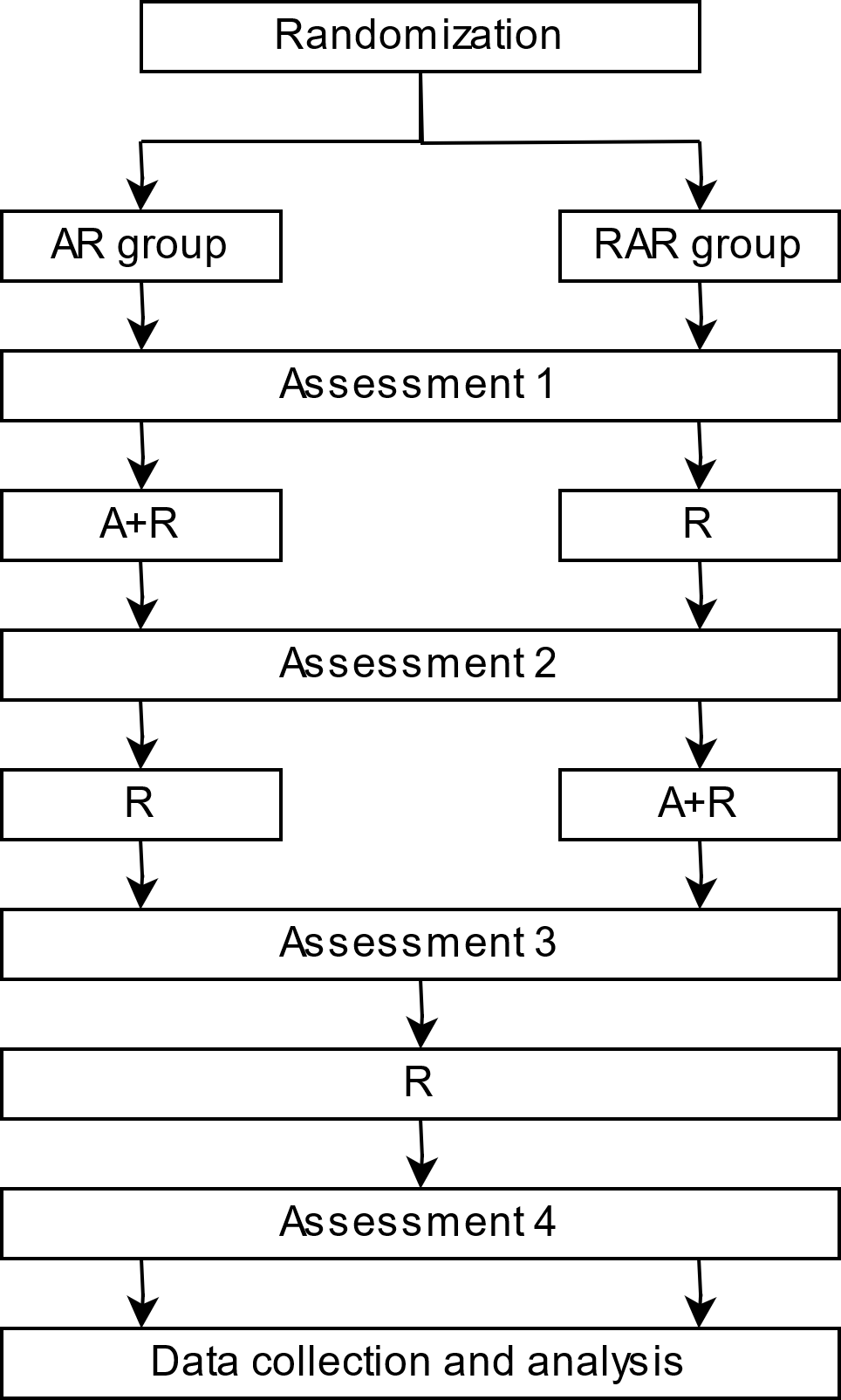


**9. Statistical analysis**

Statistical analyses were performed using SPSS 24.0 (IBM Corporation, Armonk, NY, USA) software, we followed the statistical analysis referenced from the protocol we refer-enced, We analyzed the data of four effects (treatment, period, carry-over, and follow-up ef-fects) to assess the effects of the RAGT period on GMFM-88, PBS, 6MWT, PCI, and EVGS.


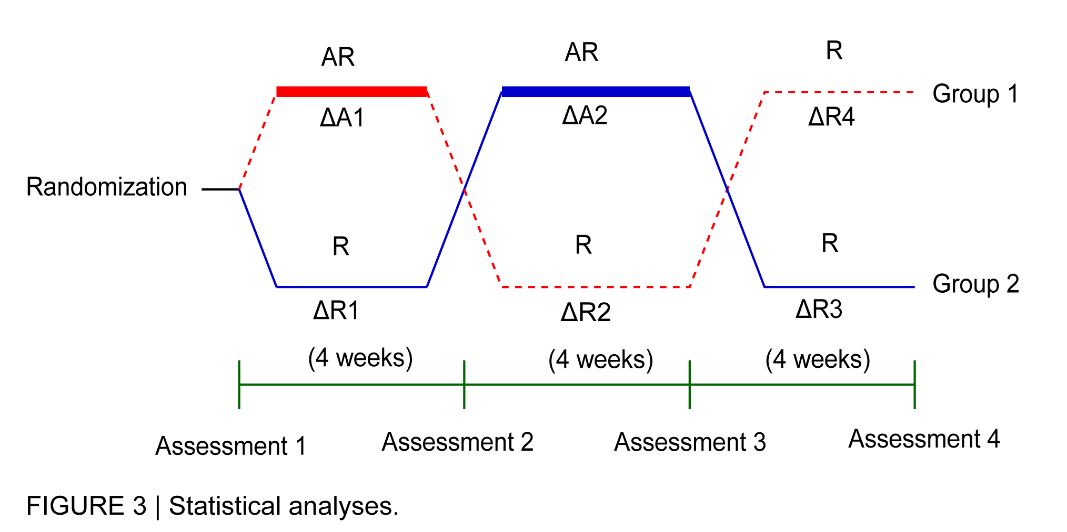


Figure 3

Treatment effects were compared using delta (Δ) values obtained during AiWalker-K (A) and routine rehabilitation training (R) (ΔA1 grouped with ΔA2 vs ΔR1 grouped with ΔR2). Period effects were compared using the Δ values obtained during the first and second training sessions (ΔA1 grouped with ΔR1 vs ΔA2 grouped with ΔR2). Carry-over effects were compared using the sum of the delta (Δ) values (ΔA1+ΔR2 vs ΔR1+ΔA2) for the first two periods between Group 1 and Group 2. Finally, follow-up effects were compared us-ing a fourth assessment of Group 1 and Group 2. Between-group differences in heart rate, blood pressure, baseline, and follow-up effects were analyzed using independent t-tests, Mann-Whitney U tests, chi-square for binary variables, or Fisher's exact test. The paired t-tests or Wilcoxon’s signed-rank tests were used to analyze treatment, period, and carry-over effects, respectively. Finally, independent t-tests or Mann-Whitney U tests were used to determine differences between groups. The level of statistical significance for all tests was set at P< 0.05. As shown in Figure 3.

The training sequence of Group 1 was AR training→R training→R training, while the training sequence of Group 2 was R training→AR training→R training. Delta (Δ) repre-sents the increment. The training content for ΔA1 and ΔA2 included AiWalker-K training (A) combined with routine rehabilitation training (R), while the training content for ΔR1, ΔR2, ΔR3, and ΔR4 was routine rehabilitation training (R).

**10. Safety analysis**

The equipment used in this experiment is an AiWalker-K children's lower limb movement rehabilitation robot developed by Beijing Da Ai Robot Technology Co., Ltd., which has been certified by relevant institutions: [Product Technical Requirements No.] Beijing Machinery Registration No. 20182260229; 【 Production Enterprise License Number 】 Jingshi Pharmaceutical Supervision Instrument Production License No. 20180027; Product Registration Certificate No.: JXZZ No.20182260229. AiWalker K is safe and reliable in its working principle. During the children's wearing and training period, it will be accompanied by trained therapists and parents throughout the entire process. The children will receive training in the pre trial preparation stage. During the training period, the child is in the equipment, and the stability of the equipment is maintained by the soles of the feet and mobile platform. At the same time, there are dedicated personnel behind to control and operate the equipment to ensure the safety of the child. After wearing the device, the child may experience minor adverse reactions such as skin abrasion, contusion, limb numbness, and pain. Although the probability of occurrence is extremely low, once these adverse reactions occur, immediately stop training and provide targeted treatment, and closely observe.

Equipment introduction: length 870mm × Width 825mm × 1140mm high, suitable for children with a height of 80cm~150cm, weight ≤ 70kg, and lower limb motor dysfunction, mainly characterized by lumbar fixation, lower limb drive, and true ground walking. The device mainly consists of a control system, a drive system, and a mobile platform. The control system is responsible for outputting motion gait, coordinating synchronous and coordinated movements among the four drivers, and adjusting the motion parameters of the drivers in real-time based on the angular velocity, angular velocity, and other state information of the motor shaft. The rotation centers of the bilateral hip and knee joint motors in the driving system are on the same horizontal axis as the user's hip and knee joint rotation centers. The thigh and lower leg rods are adjustable structures, with convenient and accurate length adjustment, which can quickly adapt to users of different heights and body types. At the same time, the rotation centers of the bilateral hip and knee joint motors and the initial angle formed by the large and small leg rods can be adjusted. There are handrails behind the mobile platform and four wheels below, making it easy to move the device. In addition, the device is connected to the wearer's limbs through straps on the waist, thighs, calves, and feet. In addition, the distance between the two moving connection sections of the lumbar support device is adjustable, suitable for users with different waist widths. At the same time, lower limb movements can drive the lumbar support device to move up and down relative to the predetermined position. The maximum range of motion of the AiWalker K joint is: hip joint flexion not less than 90 ° and extension not less than 30 °; Knee joint flexion not less than 100 ° and extension not less than 5 °; Ankle dorsiflexion should not exceed 30 degrees. The gait cycle can be adjusted between 2.45 seconds and 5.25 seconds as needed.

**11. Feasibility analysis**

Before children and parents participate in the experiment, the working principles and technical indicators of AiWalker K will be introduced to parents by staff and therapists. During the use of AiWalker K by children, the entire process is accompanied and guided by trained therapists and staff. Familiarity training will be conducted during the pre trial preparation phase, and then staff or therapists will slowly increase equipment assistance to select equipment parameters that are more suitable for the patient. At the same time, children will also be accompanied and guided by trained therapists and staff during the training period. The preparation stage before the formal trial has confirmed its feasibility and good performance.

**12. Research Schedule**

| Starting and ending time | research contents |
| --- | --- |
| 2022.09-2022.10 | Literature review |
| 2022.11-2023.06 | Completing experiments and writing papers |
| 2023.07- | Paper Submission |

**13. Ethical issues involved**

The AiWalker-K used by the experimental group of children belongs to a wearable mobile rehabilitation robot, which fully adopts the assistance mode of passive machine assistance for children's walking. Its safety is high, it has passed CFDA certification, and has been recognized by multiple tertiary hospitals in the country. No additional fees will be charged for training, evaluation, and examination related to the experiment, and there will be no additional financial burden on the patient. During the experiment, professional rehabilitation therapists and the patient's family will accompany the patient throughout the entire process to ensure their safety. This project fully complies with clinical medical research standards, and all children are receiving routine rehabilitation treatment. It is necessary to be included in the study after the family members are fully informed and sign an informed consent form, and can voluntarily withdraw midway to ensure the voluntary principle of the children and their families. No additional ethical issues are involved.

**Informed consent process**

The physician in charge of rehabilitation medicine or above shall inform the family members of the benefits and risks of this study. Parents (parents or grandparents) shall confirm and sign the informed consent form, and shall not apply for exemption from informed consent.

Applicant's signature:

Date: MM/DD/YYYY

**Attachment 1**

**AiWalker K device operating instructions:**

**1. Measure the limb size of the child:**

A Measurement tool: It is recommended to use a tape measure;

B Pelvic width: measure the distance between the greater trochanters on both sides;

C Thigh size: Measure the distance between the hip joint axis and the knee joint axis;

D calf size: measure the distance between the knee joint axis and the sole of the shoe;

E Waist depth dimension: measure the farthest distance from the midline of the human body to the buttocks in the sagittal plane;

Record the above information in a book.

**2. Adjust the device size:**

A Adjust the height of the lumbar device;

B Adjust the waist width of the device;

C Adjust the waist depth of the device;

D Adjust the size of the equipment leg to ensure that the length of the equipment leg is consistent with the length of the child's leg.

**3. Wearable devices:**

A Shake the height adjustment handle to allow the foot pedal to leave the B ground at a certain distance, ensuring that the foot pedal does not touch the ground when the equipment is standing;

C Shake the height adjustment handle to lower the foot pedal to the ground, shake the height adjustment handle to slowly lower the lumbar device, and at the same time, slowly pull up the mobile platform backwards until the device bends the hips and knees, and the hip joint sits on the support stool. Press the brake pedal down and lock the casters;

D Untie the straps on the device and allow the child to sit on the device with both feet on the foot pedals;

E Finally, tighten the straps and fix the child's waist, shoulders, soles, and waist in the corresponding positions of the device to complete the wearing of the device. (Recommended wearing sequence: waist strap - shoulder strap - plantar strap - calf strap - thigh strap).

**4. Control operation:**

A Before standing up, the child should wear the equipment and maintain a sitting position. Grasp both ends of the mobile platform armrest with both hands, and the operator should shake the height adjustment handle on the mobile platform to gradually raise the hip joint, causing the device's soles to leave the ground, ensuring that the soles of the feet do not touch the ground when the device is standing;

B Turn on the power switch and wait for the system to load. Follow the screen prompts, add or select a child, and then enter the training interface;

C Set corresponding training parameters (including initial angle, gait selection, scaling ratio, gait cycle, training time, etc.);

D Click the "Reset" button, the device will reach the reset state, and then slowly lower the height of the device to make the distance between the foot of the device and the ground about 2cm;

E Click the "Start" button to start rehabilitation training. The operator shakes the height adjustment handle to gradually touch the device's foot to the ground, and the page displays a countdown;

F If it is necessary to stop walking, first use the height adjustment handle to lift the child off the ground (preferably with the distance between the hip joint and the ground greater than the leg length), then double-click the countdown page, keep the device in its current position, and then click the "reset" button to restore the device to an upright position; Finally, use the height adjustment handle to lower the equipment to the ground;

G If it is necessary to sit down after stopping, the operator presses the power switch and the equipment will be disconnected from the power supply. At this time, the operator needs to lower the equipment through the height adjustment handle while pulling the frame to move back. Under the action of gravity, the equipment will appear in a sitting position; Finally, use the height adjustment handle to lower the equipment to the ground;

H If an emergency situation requires an emergency stop, quickly press the emergency stop button to cut off the power supply of the equipment. When conducting further training, the emergency button needs to be turned up;

I If you want to end the training, please operate the device to make the child sit in a sitting position. The process of sitting is shown in f and g Then loosen all straps to transfer the child.

**References**

1. [] Oskoui M, Coutinho F, Dykeman J, et al. An update on the prevalence of cerebral palsy: a systematic review and meta‐analysis[J]. Dev Med Child Neurol, 2013, 55(6): 509-519. [↑](#endnote-ref-1)
2. [] Gulati S, Sondhi V. Cerebral palsy: an overview[J]. Indian J Pediatr, 2018, 85(11): 1006-1016. [↑](#endnote-ref-2)
